# Impact of Incorporating Nanoparticles to Adhesive Resin on the Demineralization of Enamel: A Systematic Review

**DOI:** 10.3390/dj13030089

**Published:** 2025-02-20

**Authors:** Naif Almosa

**Affiliations:** Department of Paediatric Dentistry and Orthodontics, College of Dentistry, King Saud University, Riyadh 11545, Saudi Arabia; nalmosa@ksu.edu.sa

**Keywords:** nanoparticles, nanotechnology, adhesives, adhesion, enamel, demineralization

## Abstract

**Background/Objective:** Many novel solutions for a range of dental problems are emerging as a result of the quick development of nanotechnology and nanocomplex synthetic techniques. The effectiveness, quality, and negative consequences of these advancements are occasionally debatable, though. This systematic review sought to better summarize the existing additions of nanoparticles to dental adhesive systems in order to improve their performance and properties, evaluate their quality, and examine the results that have been published. **Materials and methods:** The present systematic review was carried out according to PRISMA guidelines. The search was carried out on PubMed central, Cochrane collaboration, Science direct and Scopus scientific engines. Selected MeSH keywords (nanoparticles, adhesive resin, enamel demineralization) were used for data extraction. A total of 13 full-text original articles were included in the final analysis, and these articles were based on adding nanoparticles to the adhesive resin to evaluate their effects on enamel demineralization. **Results:** The literature search resulted in a total of 13 original studies/articles up until November 2024. The text articles comprised in vitro studies with robust inclusion and exclusion criteria. The review included various types of adhesives and nanoparticles, with amorphous calcium phosphate (ACP) being the most common. Other nanoparticles included polydopamine–Ag, bioactive glass, and silver. Most studies assessed the effects of nanoparticles on adhesive shear bond strength (SBS), microbial growth, and microhardness. Only three studies investigated the effects of nanoparticles on microhardness using Vickers tests. **Conclusions:** The review found that adding nanoparticles to orthodontic dental adhesives enhances their antibacterial and anticariogenic properties without affecting the shear bond strength. This could prevent enamel demineralization during orthodontic therapy. Future research could benefit from these positive properties, necessitating an interdisciplinary approach.

## 1. Introduction

Tiny particles with sizes ranging from 1 to 100 nanometres (nm), where 1 nm is one-billionth of a meter, are called nanoparticles. Nanoparticles have distinct physical and chemical characteristics that set them apart from their bulk counterparts because of their small size [1]. Because of their high surface area to volume ratio, nanoparticles are more reactive and interact better with other materials. Quantum effects become important at the nanoscale, affecting the optical, electrical, and magnetic characteristics of nanoparticles. Metals, ceramics, polymers, and composites are just a few of the materials that can be used to create them [2].

Because of their special qualities at the nanoscale, nanoparticles provide numerous advantages in a variety of industries. Drug delivery methods can be enhanced by nanoparticles, increasing the efficacy and precision of treatments. For instance, they can minimize harm to healthy cells by delivering chemotherapy medications straight to cancer cells. Materials can become stronger, lighter, and more resilient by incorporating nanoparticles [3,4]. The sports equipment, automobile, and aerospace industries can all benefit from this. Electronic gadgets may now be produced more quickly, efficiently, and in smaller sizes thanks to nanotechnology. This covers anything from sophisticated computer systems to cell phones. Water filtration systems can employ nanoparticles to more efficiently remove impurities. Additionally, they contribute to energy conversion and storage, as shown in more effective solar panels and batteries [5]. The addition of nanoparticles improves commonplace products like antimicrobial surfaces, scratch-resistant coatings, and stain-resistant textiles. Nanoparticles can function as catalysts in chemical reactions to quicken them, increasing industrial operations’ efficiency and lowering the requirement for harsh chemicals [6].

Because of their potent antibacterial qualities, nanoparticles—like silver nanoparticles—are utilized in medical device coatings and wound dressings to help stop infections. To encourage quicker and more efficient wound and burn healing, nanoparticles can be added to creams and dressings [7]. Zinc oxide nanoparticles and natural extracts, for instance, have demonstrated encouraging outcomes in the treatment of skin burns [8]. Innovative immunological treatments for illnesses like cancer are being developed using nanoparticles. In order to better identify and combat cancer cells, they can aid in immune system modulation [3,4]. To treat genetic problems, nanoparticles can introduce genetic material into cells. This approach is being investigated for the treatment of muscular dystrophy and cystic fibrosis [9]. These uses demonstrate how nanoparticles can revolutionize medical interventions and enhance patient care.

**Figure 1:**
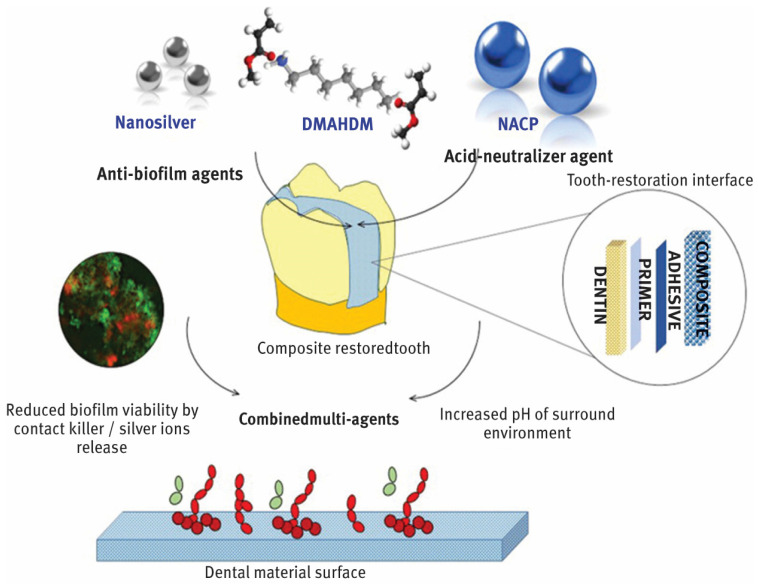
Incorporation of AgNPs into dental structure. From “Designing Multiagent Dental Materials for Enhanced Resistance to Biofilm Damage at the Bonded Interface”, by Melo, MA et al. [10], ACS Applied Materials & Interfaces 2016 8 (18), 11779–11787 copywritted by ACS publications [10].

In dentistry, nanoparticles are advancing significantly and providing creative fixes for a range of tooth problems. By blocking the growth of dangerous bacteria, nanoparticles with potent antibacterial qualities, including zinc oxide and silver, help to prevent dental caries or tooth decay as shown in Figure 1 [10,11,12]. In order to improve the treatment of gum disorders, periodontal therapy can employ nanoparticles to transport medications straight to the afflicted areas [13]. Implants and prostheses can be made more resilient to wear and strain by adding nanoparticles to dental materials to improve their mechanical qualities. In order to increase the strength, durability, and aesthetic appeal of fillings and restorations, nanocomposites are utilized [14]. Better adhesion and fracture resistance are offered by these materials [15]. The early diagnosis of dental disorders is made possible by nanoparticles, which increase the sensitivity and precision of diagnostic instruments like imaging techniques [16]. To lower the risk of infections and enhance general hygiene in dental offices, nanoparticles can be applied to dental tools and surfaces. Dental nanotechnology, sometimes known as “nanodentistry”, is constantly developing and holds the promise of more effective and efficient dental treatment options down the road [10,11,12,13,14,15,16].

Recent research has focused on adding various nanoparticles to dental adhesives to improve their physical characteristics and antibacterial capabilities [11,12]. Elastomeric ligatured supported NPs like Benzocaine and silver have shown anticariogenic and antimicrobial effects, preventing dental decay and promoting oral health. Combining silver NPs with zinc oxide and chlorhexidine enhances tensile strength and anti-inflammatory properties, improving orthodontic material durability [16]. Gold nanoparticles have shown improved antibacterial properties, preventing enamel demineralization and maintaining adhesive integrity [13,14]. Titanium dioxide NPs enhance antibacterial efficacy without compromising adhesive shear bond strength [15]. Calcium phosphate NPs are stable and cost-effective, inhibiting bacterial growth and making them an attractive option for future development in dental adhesives [16,17].

The rationale for a review study on the application of nanoparticles in dental adhesives stems from the growing body of evidence that demonstrates the wide-ranging benefits of incorporating NPs into dental materials in addition to the limited evidence on how this affects enamel demineralization. By synthesizing and evaluating these studies, the present review aims to provide a comprehensive understanding of the mechanisms behind the enhanced properties of nanoparticle-enhanced adhesives, while also highlighting the potential for further innovation in the field. The growing interest in antimicrobial, mechanical, and anti-inflammatory effects makes this an important area of research with the potential to significantly improve dental care outcomes, particularly in orthodontics and preventive dentistry.

## 2. Material and Methods

This research project has been recognized and approved by the College of Dentistry Research Center (CDRC) at King Saud University, with registration number FR 0723.

### 2.1. Research Question

In this systematic review, we followed preferred reporting items from the systematic review and meta-analysis (PRISMA) guidelines. The main question was “Is there any impact of incorporating nanoparticles to adhesive resin on the demineralization of enamel?”

### 2.2. Search Strategy

**Figure 2:**
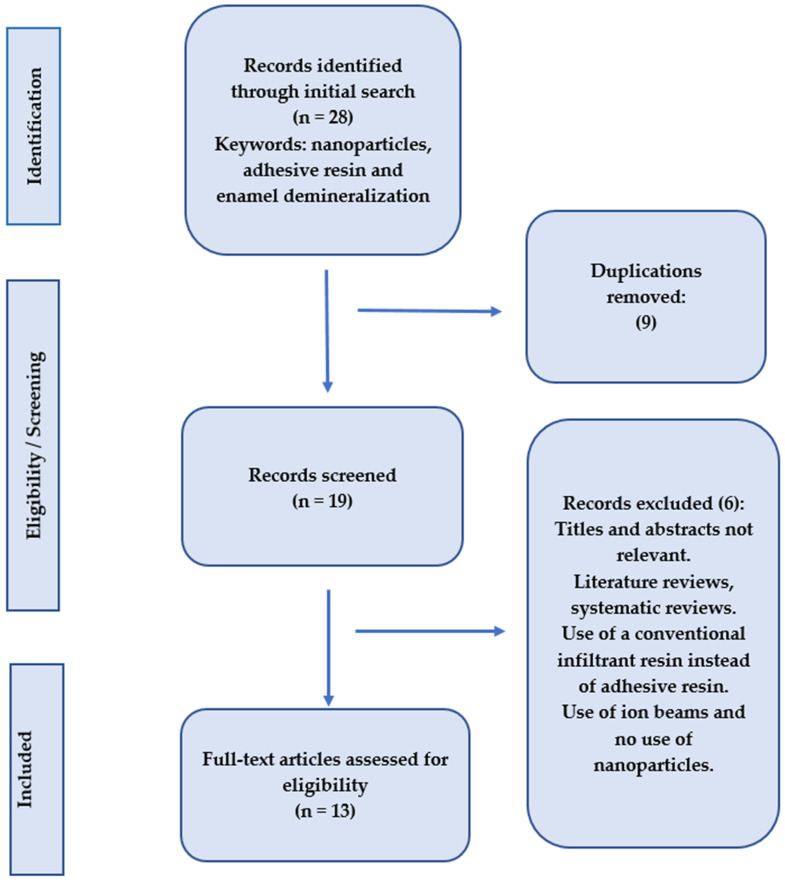
Search methodolgy employed in the present systematic review.

The PubMed Central, Cochrane Collaboration, Science Direct, and Scopus databases were searched for abstracts and full texts of papers, related to the subject, that were accessible online until November 2024, without regard for language limitations, as part of this systematic review. Keywords associated with “adhesive resin”, “enamel demineralization”, and “nanoparticles” were the main focus of the search. By carefully examining the references from the included publications, the search was further broadened. After carefully gathering all pertinent information, the principal investigator examined the findings. Only original research articles with full texts were included; after reviewing the abstracts and titles, any repeated studies were eliminated (Figure 2).

### 2.3. Eligibility Criteria for Literature Search

The following criteria were utilized to evaluate each included article and extract the data: study design, adhesive systems used, authors’ names, journal and year of publication, study outcomes, and nanoparticles added to the adhesives (Table 1 and Table 2).

### 2.4. Exclusion Criteria

After reviewing the titles and abstracts, studies that were surveys, clinical reports, literature reviews, or systematic reviews were eliminated. Studies with insufficient information or those that did not concentrate on adding nanoparticles to adhesives were not included in our review (Table 3).

## 3. Results

### 3.1. General Characteristics of Included Studies

All studies included were in vitro research studies.

Different types of adhesives and nanoparticles were used in the studies included in the review. The most common adhesive used was Transbond™ XT (Transbond™ XT Light Cure Adhesive, 3M, Unitek, Dubai, UAE), which is typically used to bond metal and ceramic brackets to enamel surfaces. Six studies included in the review used this adhesive. Other adhesives used in the studies were Scotchbond™ (3M™ Scotchbond™ Universal Plus Adhesive, 3M, Unitek, Dubai, UAE), Clearfil S3 Bond™ (CLEARFIL™ S³ BOND PLUS, Kuraray, Noritake, Europe GmbH, Hattersheim, Germany), Adper Single Bond 2™ (3M™ Adper™ Single Bond 2, 3M, North Ryde, NSW, Australia), and Ortho Connect™ (GC Ortho Connect™, GC Orthodontics, Alsip, IL, USA).

The most prevalent nanoparticle added to adhesives in the trials included in the review was amorphous calcium phosphate (ACP), which was used in four investigations. Polydopamine-Ag, bioactive glass containing 2.5% fluoride, quaternary ammonium resin monomer, arginine-loaded mesoporous silica, silver and silver diamine fluoride, nano-hydroxyapatite, silica nanofillers, titanium oxide (TiO_2_), nano-bioactive glass–silver and Emodin were some of the other nanoparticles used in different studies.

Most of the studies looked at how adding nanoparticles to adhesives affected the shear bond strength (SBS) of the adhesive to enamel, with 11 studies testing SBS. Nine studies tested the effects of nanoparticles on microbial growth using colony counting and cell viability tests to check for cytotoxicity. Only three studies examined the impacts of nanoparticles on microhardness, using the Vickers test.

### 3.2. General Outcomes of Included Studies

Most studies found that adding nanoparticles to adhesives did not significantly affect the shear bond strength (SBS) between the adhesive and enamel, but it did improve the anti-cariogenic properties. Amorphous calcium phosphate (ACP) was added as a nanoparticle in many of the studies, and the SBS was similar to that in the control group [16,17,18,19]. In one study by Jia A et al. (2023) [17], the SBS of an adhesive with 0.2% amorphous calcium phosphate–polydopamine–silver nanoparticles was 11.89 ± 1.27 MPa, which achieved the minimum clinical bond strength of 7.8 MPa. The anti-demineralization test showed that adding 2.5% fluoride bioactive glass as a nanomaterial to the adhesives helped prevent enamel demineralization. When exposed to fluorescent mineralizing adhesives, both demineralized enamel and dentin, along with reconstituted type I collagen, could undergo remineralization at the extra- and intra-fibrillar levels [20].

Adhesives with arginine-loaded mesoporous silica nanoparticles showed significantly stronger antibacterial activity and better acid inhibition without compromising bonding strength or biocompatibility, compared to conventional adhesives [21]. When silver nanoparticles and silver diamine fluoride nanoparticles were added, there was no noticeable difference in performance compared to adhesives without these anti-caries compounds. However, adding silver nanoparticles improved the microhardness of the teeth. These antimicrobial compounds could potentially be used as a pretreatment to prevent caries before resin restoration [22,23]. Additionally, adding hydroxyapatite nanoparticles to orthodontic composites increased the mineral content and microhardness of surrounding enamel, but the shear bond strength (SBS) decreased as the nanoparticle amount increased [24,25].

Experimental composite adhesives with silica nanofillers and silver nanoparticles showed no significant difference in shear bond strength (SBS) or bond failure compared to regular adhesives [26]. Twenty-four hours after curing, there was no noticeable difference in SBS between regular adhesives and those with titanium oxide (TiO_2_) nanoparticles (*p* = 0.58). However, the TiO_2_ nanoparticles showed better antibacterial activity than the regular adhesives, with a significant difference between the two groups (*p* = 0.03) [27]. The 2% concentration of Emodin nanoparticles also performed much better than the control group in fighting *S. mutans* (*p* < 0.05) [28].

### 3.3. Risk of Bias Assessment

**Figure 3:**
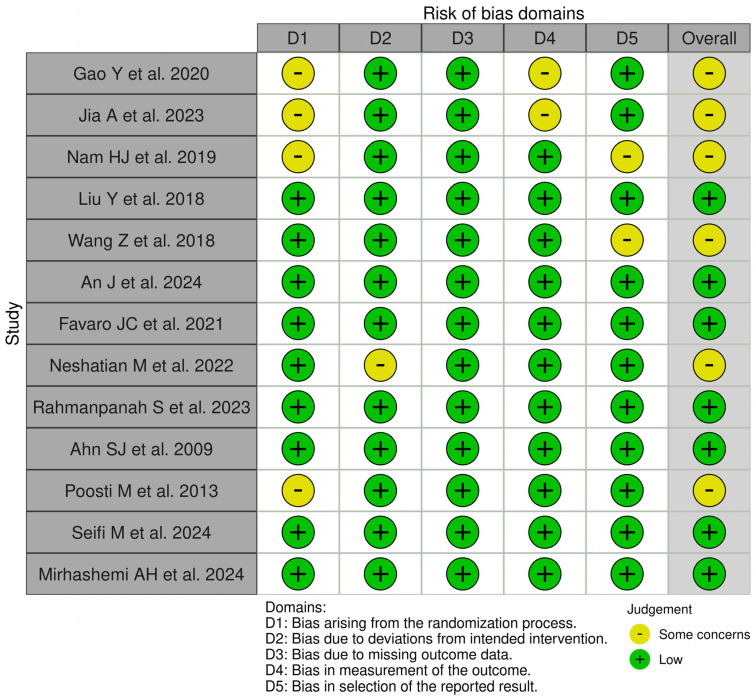
Qualitative analysis of the included studies by Robvis© (Cochrane Methods Bias, The Cochrane Collaboration, London, United Kingdom) [16,17,18,19,20,21,22,23,24,25,26,27,28].

Figure 3 displays the risk of bias assessments for the studies included in this systematic review. The findings reveal that while some of the investigations raised concerns and had the potential for bias, the majority of the studies included had a low risk of bias. In general, all included studies had a low risk of bias.

## 4. Discussion

In recent years, nanotechnology has evolved, and nanoparticles have been added to several materials to enhance their mechanical as well as biological properties [10,11,12,13,14,15]. Dental adhesives are being enriched with nanoparticles to improve their performance and properties [16,17]. Nano-hydroxyapatite and silica penetrate dentinal tubules, creating stronger bonds and reducing bond failure risk [24]. They minimize shrinkage during polymerization, preventing gaps and secondary caries. Nanoparticles also enhance mechanical properties like elastic modulus and tensile strength, making them more durable. Some nanoparticles, like silver and zinc oxide, have antibacterial properties, preventing bacterial growth and infections [22]. Nano-hydroxyapatite releases fluoride ions, remineralizing tooth structure and preventing decay. Different types of nanoparticles include nano-hydroxyapatite, silica, silver, and zinc oxide [14]. These advancements make dental treatments more effective and long-lasting. Therefore, this systematic review was conducted to analyze and compare the existing research articles on the additions of nanoparticles to dental adhesive systems in order to improve their performance and properties, evaluate their quality, and examine the results that have been published.

In orthodontics, braces come in a variety of forms. Compared to bands, bonded brackets offer a number of benefits, including improved appearance, simplicity in placement, and accessibility and removal for dental care [34]. Although it is challenging to clean by regular brushing, the bracket adhesive–enamel junction is the most common location for bacterial adherence and biofilm formation [35]. White spots, tooth cavities, and enamel decalcification are frequent outcomes of the plaque buildup surrounding orthodontic brackets [36]. Additionally, eliminating microbial growth surrounding orthodontic appliances is challenging. The material of the bracket, the design of the orthodontic brackets, and the ligating technique all play significant roles in its adherence to the permanent appliance. Surface roughness and surface-free energy are two of the many variables that affect plaque quantity and quality [34,35,36]. Microorganisms’ adherence to surfaces is also influenced by van der Waal forces and electrostatic attractions [37].

Dental adhesives are enhanced by nanoparticles, which improve their properties through various mechanisms. These nanoparticles, like silica, titanium dioxide, or hydroxyapatite, act as fillers in the adhesive matrix, enhancing its stiffness, tensile strength, and durability. They create a more uniform bond between the adhesive and the enamel/dentin surface, promoting better adhesion [38,39]. Nanoparticles like silver, copper, and titanium oxide have natural antimicrobial effects, reducing bacterial growth and preventing decay and inflammation. Some nanoparticles, like amorphous calcium phosphate (ACP), release calcium and phosphate ions, protecting enamel from demineralization. They also promote the remineralization of the enamel by releasing minerals that restore mineral content in demineralized areas, strengthening the tooth structure [40]. Nanoparticles like hydroxyapatite or bioactive glass increase the microhardness of both the adhesive and the surrounding enamel, enhancing wear resistance and bond strength. Some nanoparticles are biocompatible and less toxic, reducing the risk of adverse reactions in the oral cavity and improving the overall safety of the adhesive [41]. Nanoparticles, typically 1–100 nm, have a higher surface area, enhancing bonding strength and antibacterial activity. Their material, like silver, titanium dioxide, or hydroxyapatite, determines their effect. The concentration of nanoparticles must be controlled to ensure desired properties. Even dispersion in the adhesive matrix is crucial for consistent performance. Modifying the surface of nanoparticles can improve compatibility with the matrix. The pretreatment of the tooth surface, specific properties of enamel and dentin, and curing processes also impact nanoparticle integration. The oral environment’s pH and moisture can also affect nanoparticle stability and effectiveness [1,2].

The demineralization of enamel can be considerably impacted by the addition of nanoparticles to adhesive resins. Research has indicated that the characteristics of resin infiltrants can be improved by the addition of nanoparticles, such as nano-silica (NS) [26]. For example, it has been discovered that adding NS to resin infiltrants increases the mineral density of demineralized enamel while simultaneously decreasing water sorption and solubility. As a result, the resin is better able to penetrate and stabilize the enamel, possibly stopping additional demineralization [26]. Furthermore, it has been demonstrated that adding amorphous calcium phosphate, silver nanoparticles, hydroxyapatite nanoparticles and titatium dioxide (TiO_2_) to adhesive resins improves their cytocompatibility and maintains clinically acceptable shear bond strength levels [16,17,18,19,20,21,22,23,24,25,26,27,28]. This implies that these changes can enhance dental adhesives’ overall functionality and robustness, increasing their ability to shield enamel from demineralization.

Glucans and a variety of bacteria, the most prevalent and most cariogenic of which is *Streptococcus mutans* (*S. mutans*), make up adult dental plaque [42,43]. Researchers have investigated the connection between *S. mutans* CFUs on the surfaces of several orthodontic material types. The adherence of *S. mutans* to ceramic, plastic, and stainless steel brackets was not significantly different, according to research by Juvvadi et al. [44], Fournier et al. [45], Papaioannou et al. [46] and Brusca et al. [47]. The findings of Ahn et al. were very different [48]. Compared to ceramic and plastic brackets, stainless steel brackets had more CFUs. Compared to stainless steel brackets, titanium and gold brackets displayed fewer CFUs. A lower number of CFUs *S. mutans* was found in gold brackets, which may be due to the inert qualities of gold [44]. Because they have the highest critical surface tension (more surface energy), metallic brackets have a higher level of bacterial adherence than ceramic brackets. Microorganisms were more likely to adhere to stainless steel. When used properly, a substance with a high surface free energy will draw more germs than one with a low one [44,47,48].

The ability of orthodontic adhesives to hold onto cariogenic streptococci was greater than that of bracket materials [48]. When orthodontic brackets were fixed with nano-filled adhesives, prior short-term (24 h) in vitro investigations showed equivalent or lower (but nevertheless adequate) shear strength [49]. The use of nanofillers decreased the adhesive’s surface roughness in comparison to conventional orthodontic adhesives; however, this was not the case when silver nanoparticles were added to the combination. However, it has not yet been determined how well adhesives with nanofillers work over the long run to stop enamel demineralization during orthodontic treatment, especially under orthodontic bands and around brackets [50].

The contact between the polymeric resin and the tooth can deteriorate due to bacterial acids, microleakage, and cyclic stressors. It is a challenging and thought-provoking task to conduct research on the integration of state-of-the-art antimicrobial agents for the creation of novel, durable, bioactive resin-based dental materials [37]. When added to dental materials like dental adhesives, the antibacterial agents released, such as silver nanoparticles, amorphous calcium phosphate nanoparticles, titanium oxide nanoparticles, and several others, have demonstrated promise [16,17,18,19,20,21,22,23,24,25,26,27,28]. Melo and colleagues [51] conducted a thorough examination of the synthesis of silver nanoparticles and their potential incorporation into dental primers, adhesives, and composites, all of which are necessary for performing composite restoration [51].

Most of the studies in the present review were in vitro studies, and the results were not correlated with in vivo investigations. None of the publications included data from human clinical trials that would have indicated the effectiveness of the adhesives with nanoparticles used in orthodontics. The usage of dental adhesives in clinical settings has expanded due to the development of nanotechnologies, which promote contact between nanoparticles and oral microbiota. We suggest that more researchers look into and address clinical trials, in vivo studies and clinical correlation to predict the actual outcomes of the functional uses of dental adhesives loaded with nanoparticles in dental clinics, especially orthodontic clinics, in order to examine these limitations and strengthen the scientific evidence.

## 5. Conclusions

According to the review’s findings, adding nanoparticles to orthodontic dental adhesives improved their antibacterial and anticariogenic qualities while having no discernible impact on the shear bond strength between the tested dental adhesives and enamel. In order to stop enamel demineralization during orthodontic therapy, nanoparticles can be effectively added to orthodontic adhesives. Future orthodontics research could be greatly aided by the recent development of positive antibacterial properties. The effectiveness of orthodontic therapy has increased due to the physicochemical characteristics that nanomaterials have acquired. It is expected that the use of nanomaterials in dentistry, particularly in orthodontics, will increase further, necessitating an interdisciplinary approach that emphasizes knowledge of both dentistry and nanomaterial research.

## Figures and Tables

**Table 1:** General characteristics of selected studies included in the systematic review.

No.	Authors	Journal. Year	Study Design	Study Title	Assessment Method
1.	Gao Y et al. [16]	Journal of Dentistry. 2020	In Vitro	Enamel remineralization via poly(amido amine) and adhesive resin containing calcium phosphate nanoparticles.	* SBS
2.	Jia A et al. [17]	Journal of Functional Biomaterials. 2023	In Vitro	Developing a Novel Enamel Adhesive with Amorphous Calcium Phosphate and Silver Nanoparticles to Prevent Demineralization during Orthodontic Treatment.	SBS and Colony Counting Test
3.	Nam HJ et al. [18]	Materials (Basel). 2019	In Vitro	Fluorinated Bioactive Glass Nanoparticles: Enamel Demineralization Prevention and Antibacterial Effect of Orthodontic Bonding Resin.	Vicker’s Test and Cell Viability Tests
4.	Liu Y et al. [19]	Journal of Dentistry. 2018	In Vitro	Antibacterial and remineralizing orthodontic adhesive containing quaternary ammonium resin monomer and amorphous calcium phosphate nanoparticles.	SBS and Cell Viability Tests
5.	Wang Z et al. [20]	Nanoscale. 2018	In Vitro	A novel fluorescent adhesive-assisted biomimetic mineralization.	Cell Viability Tests
6.	An J et al. [21]	Journal of Dentistry. 2024	In Vitro	Formulation of arginine-loaded mesoporous silica nanoparticles (Arg@MSNs) modified orthodontic adhesive.	SBS and Cell Counting Kit
7.	Favaro JC et al. [22]	Restorative Dentistry & Endodontics. 2021	In Vitro	Can silver diamine fluoride or silver nanoparticle-based anticaries agents affect enamel bond strength?	SBS and Microhardness Test
8.	Neshatian M et al. [23]	Materials Today Bio. 2022	In Vitro	Promoting mineralization at biological interfaces Ex vivowith novel amelotin-based bio-nano complexes.	Mineral Formation and Sbs Testing
9.	Rahmanpanah S et al. [24]	International Orthodontics. 2023	In Vitro	Evaluation of shear bond strength and enamel remineralizing effect of experimental orthodontic composite containing nano-hydroxyapatite: An in vitro study.	SBS
10.	Ahn SJ et al. [25]	Dental Materials. 2009	In Vitro	Experimental antimicrobial orthodontic adhesives using nanofillers and silver nanoparticles.	SBS and Bacterial Adhesion Assay
11.	Poosti M et al. [26]	European Journal of Orthodontics. 2013	In Vitro	Shear bond strength and antibacterial effects of orthodontic composite containing TiO_2_ nanoparticles.	SBS and Antibacterial Tests
12.	Seifi M et al. [27]	BMC Oral Health. 2024	In Vitro	Investigation of mechanical properties, remineralization, antibacterial effect, and cellular toxicity of composite orthodontic adhesive combined with silver-containing nanostructured bioactive glass.	Cytotoxicity Test, SBS and Microhardness Tests
13.	Mirhashemi AH et al. [28]	International Orthodontics. 2024	In Vitro	In vitro effects of antimicrobial properties and shear bond strength of different concentrations of Emodin nanoparticles incorporated orthodontic composites.	Antimicrobial Activity and SBS Tests

* SBS = Shear bond strength.

**Table 2:** Types of the nanoparticles incorporated, study outcomes and conclusions of the studies included in the systematic review.

No.	Authors	Adhesive	Nanoparticles	Study Outcomes	Conclusions
1.	Gao Y et al. [16]	Scotchbond Multi-Purpose	^a^ ACP	Adhesives with nanoparticles of ACP yielded a similar ^b^ SBS to control.	The novel SN15-PAMAM + NACP adhesive method could achieve 90% higher enamel remineralization.
2.	Jia A et al. [17]	Transbond XT	ACP and polydopamine-Ag (NPA)	SBS of adhesive with 0.2 wt. % NPA was 11.89 ± 1.27 MPa, meeting the minimal clinical bond strength of 7.8 MPa.	Adhesive with NPA may have a good application potential for the prevention and treatment of ^c^ WSL.
3.	Nam HJ et al. [18]	Transbond XT Low Flow (LV)	Bioactive glass containing 2.5% fluoride	The anti-demineralization test showed a concentration-dependent increase.	Adhesive containing bioactive glass 2.5% fluoride showed antibacterial and anti-demineralization effects, indicating possible WSL prevention activity.
4.	Liu Y et al. [19]	Transbond XT (TB)	Quaternary ammonium resin monomer and ACP	PND adhesive with 5% MAE-DB and 40% NACP exhibits antibacterial and remineralizing capabilities, and did not adversely affect SBS compared to commercial adhesive.	Novel adhesive containing quaternary ammonium monomer and ACP represents a promising candidate in combating enamel WSL and dental caries.
5.	Wang Z et al. [20]	Clearfil S3 Bond	1 wt. % of sodium fluorescein and 25 wt. % of polyacrylic acid-stabilized ACP	Fluorescent mineralizing adhesive could induce the extra- and intra-fibrillar remineralization of the reconstituted type I collagen, the demineralized enamel and dentin substrate.	The novel fluorescent adhesive-assisted biomimetic mineralization strategy will pave the way to design and produce anti-carious materials for the prevention of dental caries.
6.	An J et al. [21]	Transbond XT	Rginine loaded mesoporous silica nanoparticles	Adhesive containing Arg@MSNs exhibited significantly enhanced antibacterial activities and inhibitory effects on acid production compared to the commercial adhesive without compromising their bonding strength or biocompatibility.	Adhesive containing Arg@MSNs exhibits clinical benefits in preventing the demineralization of enamel surfaces around or beneath orthodontic brackets due to its enhanced antibacterial activities and acid-producing inhibitory effects.
7.	Favaro JC et al. [22]	Adper Single Bond 2, 3M ESPE	Silver nanoparticles (SNPs) and silver diamine fluoride (SDF)	There was no significant difference among the IE, IE + SNP, DE + SDF, and DE + SNP groups. The IE + SDF and DE groups recorded the highest and the lowest μ-SBS values, respectively. Adhesive-type failures were the most frequent for all treatments.	The use of anticaries agents (SDF and SNP) did not reduce the SBS values of resin composites when they are used on the intact or artificially demineralized dental enamel. Thus, the anticaries agents tested in this study can be used as a pretreatment prior to resin restoration contributing to caries prevention.
8.	Neshatian M et al. [23]	Enamel protein amelotin	Hydroxyapatite nanoparticles	Accelerated mineral formation collagen mineralization of bio- and nanocomplex-treated samples were observed in all model systems.	We have shown that AMTN-based bio- and nanocomplexes promote mineral formation on collagenous interfaces. Our findings can be the basis of new bioinspired, bio-nano materials that may improve dental restoration longevity by enhancing the stability and integrity of the dentin-composite resin interface.
9.	Rahmanpanah S et al. [24]	3M™ Transbond™ XT	Nano-hydroxyapatite	The addition of hydroxyapatite nanoparticles to orthodontic composite can increase the mineral content and microhardness of the adjacent enamel. However, increasing the amount of nanoparticles reduces shear bond strength in a decreasing trend.	An incremental increase in nanoparticles of HA can be incorporated into the composite to a certain extent, and limitations are determined by the mechanical properties (SBS) required for bracket bonding.
10.	Ahn SJ et al. [25]	Composite and resin-modified glass ionomer	Silica nanofillers and silver nanoparticles	There was no significant difference in shear bond strength and bond failure interface between the experimental composite adhesives and conventional adhesives.	Experimental composite adhesives can help prevent enamel demineralization around their surfaces without compromising physical properties.
11.	Poosti M et al. [26]	Transbond XT	titanium oxide (TiO_2_) nanoparticles	No significant difference was found between the SBS of conventional and nanocomposites, 24 h after curing (*p* = 0.58). The chi-square test showed that ARI scores of two groups were not significantly different after debonding (*p* = 0.69). Comparison of antibacterial effects between the conventional and the nanocomposite demonstrated a significant difference between two groups, with nanocomposites having a higher antibacterial activity (*p* = 0.03).	Colony count revealed no significant difference in bacterial growth immediately and 30 days after curing in the nanocomposite group. Adding TiO2 nanoparticles to orthodontic composite enhances its antibacterial effects without compromising the SBS.
12.	Seifi M et al. [27]	Orthodontic composite (Adhesive) (GC Ortho Connect, GC Orthodontics, Japan)	nano-bioactive glass–silver	The shear bond strength of the adhesives decreased significantly (*p* < 0.001) after the addition of nanoparticles, but it remained above the recommended value. The addition of nBG@Ag led to improvements in the microhardness of the teeth, although the differences in microhardness between the study groups were not statistically significant.	Adding nBG@Ag to orthodontic adhesives can be an effective approach to enhancing the antimicrobial activity and reducing enamel demineralization around the orthodontic brackets, without compromising biocompatibility and bond strength.
13.	Mirhashemi AH et al. [28]	Orthodontic composite (Adhesive) (GC Ortho Connect, GC Orthodontics, Japan)	Emodin nanoparticles	The eluted components test demonstrated that the 2% concentration of ENPs performed significantly better against *S. mutans* compared to the control group (*p* < 0.05). The highest mean SBS was observed with the 0.5% ENP concentration, while no significant differences in SBS and ARI were found among the groups (*p* > 0.05).	This in vitro study showed that the 2% concentration of ENP produced significantly improved antimicrobial activity without adversely affecting the SBS and ARI score. This would support the addition of 2% ENP to orthodontic adhesives.

^a^ ACP = amorphous calcium phosphate; ^b^ SBS = shear bond strength; ^c^ WSL = white spot lesions.

**Table 3:** General characteristics of selected studies excluded in the systematic review.

No.	Authors	Journal. Year	Study Title	Reason of Exclusion
1.	Yin IX et al. [29]	International Journal of Nanomedicine. 2020	Use of Silver Nanomaterials for Caries Prevention: A Concise Review	Review article
2.	Budi HS et al. [15]	Brazilian Journal of Biology. 2022	Study on the role of nano antibacterial materials in orthodontics (a review)	Review article
3.	Borzabadi-FA et al. [30]	Acta Odontologica Scandinavica. 2014	Nanoparticles in orthodontics, a review of antimicrobial and anti-caries applications	Review article
4.	Hanabusa M et al. [31]	Dental Materials. 2011	TEM interfacial characterization of an experimental self-adhesive filling material bonded to enamel/dentin	No nanoparticles used
5.	Kielbassa AM [32]	PLoS One. 2020	Ex vivo investigation into internal tunnel approach/internal resin infiltration and external Nano silver-modified resin infiltration of proximal caries exceeding into dentin	Use of a conventional infiltrant resin instead of adhesive resin
6.	Coutinho E [33]	Dental Materials. 2009	Ultrastructural characterization of tooth-biomaterial interfaces prepared with broad and focused ion beams	Use of ion beams and no use of nanoparticles
